# A decade of insight: bibliometric analysis of gut microbiota’s role in osteoporosis (2014–2024)

**DOI:** 10.3389/fmed.2024.1409534

**Published:** 2024-05-22

**Authors:** Zhi Qiang Luo, Ya Jing Huang, Ze Hua Chen, Chen Yin Lu, Biao Zhou, Xiang Hao Gong, Zhen Shen, Tao Wang

**Affiliations:** ^1^Department of Graduate School, Hunan University of Chinese Medicine, Changsha, Hunan, China; ^2^Department of Rheumatology, Kunming Municipal Hospital of Traditional Chinese Medicine, The Third Affiliated Hospital of Yunnan University of Chinese Medicine, Kunming, China; ^3^Department of Orthopedics, The Orthopedics Hospital of Traditional Chinese Medicine, Zhuzhou, Hunan, China; ^4^Department of Orthopedics, The First People’s Hospital of Xiangtan City, Xiangtan, Hunan, China; ^5^Department of Oncology, Hengyang Central Hospital, Hengyang, Hunan, China; ^6^Department of Rehabilitation, Kunming Municipal Hospital of Traditional Chinese Medicine, The Third Affiliated Hospital of Yunnan University of Chinese Medicine, Kunming, China; ^7^Department of Orthopedics, Kunming Municipal Hospital of Traditional Chinese Medicine, The Third Affiliated Hospital of Yunnan University of Chinese Medicine, Kunming, China

**Keywords:** gut microbiota, osteoporosis, bibliometric analysis, CiteSpace, VOSviewer, R package “Bibliometrix”

## Abstract

**Purpose:**

Osteoporosis represents a profound challenge to public health, underscoring the critical need to dissect its complex etiology and identify viable targets for intervention. Within this context, the gut microbiota has emerged as a focal point of research due to its profound influence on bone metabolism. Despite this growing interest, the literature has yet to see a bibliometric study addressing the gut microbiota’s contribution to both the development and management of osteoporosis. This study aims to fill this gap through an exhaustive bibliometric analysis. Our objective is to uncover current research hotspots, delineate key themes, and identify future research trends. In doing so, we hope to provide direction for future studies and the development of innovative treatment methods.

**Methods:**

Relevant publications in this field were retrieved from the Web of Science Core Collection database. We used VOSviewer, CiteSpace, an online analysis platform and the R package “Bibliometrix” for bibliometric analysis.

**Results:**

A total of 529 publications (including 351 articles and 178 reviews) from 61 countries, 881 institutions, were included in this study. China leads in publication volume and boast the highest cumulative citation. Shanghai Jiao Tong University and Southern Medical University are the leading research institutions in this field. Nutrients contributed the largest number of articles, and *J Bone Miner Res* is the most co-cited journal. Of the 3,166 scholars who participated in the study, Ohlsson C had the largest number of articles. Li YJ is the most co-cited author. “Probiotics” and “inflammation” are the keywords in the research.

**Conclusion:**

This is the first bibliometric analysis of gut microbiota in osteoporosis. We explored current research status in recent years and identified frontiers and hot spots in this research field. We investigate the impact of gut microbiome dysregulation and its associated inflammation on OP progression, a topic that has garnered international research interest in recent years. Additionally, our study delves into the potential of fecal microbiota transplantation or specific dietary interventions as promising avenues for future research, which can provide reference for the researchers who focus on this research filed.

## 1 Introduction

Osteoporosis is a systemic skeletal disease characterized by an overall reduction in bone mass and deterioration of bone microarchitecture, leading to decreased bone strength and a significantly increased risk of fractures (1). Osteoporosis presents a major health concern, especially among the elderly and postmenopausal women. The International Osteoporosis Foundation (IOF) estimates that over two hundred million people suffer from osteoporosis, which leads to a high number of fractures annually (2).

Critical to understanding osteoporosis is the recognition of the bone remodeling unit as the functional entity, within which the coupling of bone formation to resorption is tightly controlled. Disruption in this coupling in osteoporosis is mediated by a multifaceted interplay of biomolecules including, but not limited to cytokines (such as RANKL, OPG, and interleukins), growth factors (e.g., TGF-β and IGF-1), and hormones (including estrogen, testosterone, and parathyroid hormone) (3). These entities not only regulate the proliferation, differentiation, and activity of osteoblasts and osteoclasts but also modulate the local bone microenvironment and systemic bone metabolism.

Recent years have brought notable progress in osteoporosis treatments, including bisphosphonates, Selective Estrogen Receptor Modulators (SERMs), calcitonin, and parathyroid hormone (PTH) and its analogs, along with the newer Sclerostin inhibitors (4, 5). Focused on slowing bone resorption or boosting bone formation to increase bone density and lower fracture risks, these medications typically necessitate prolonged use and can entail side effects, with limited success in preventing or reversing bone loss (4). Additionally, not all patients experience identical therapeutic outcomes from these drugs, indicating considerable variations in individual responses.

In the multifactorial etiology of osteoporosis, recent research has zeroed in on the gut-bone axis, illuminating how gut microbiota can influence bone metabolism through immune modulation, metabolic product generation, and nutrient absorption, thereby revealing a potential avenue for regulating bone health (6–8). Short-chain fatty acids (SCFAs) such as butyrate and propionate, produced in the gut, are proven to directly engage in bone remodeling by fostering osteoblast proliferation and differentiation and curbing osteoclast formation (9, 10). Moreover, gut microbiota also indirectly modulates osteoporosis development by impacting the host’s immune system (11), notably through adjusting T cell subset balances and the host’s inflammatory state (12).

Although there has been significant progress in understanding the role of the gut microbiota in osteoporosis, current research still lacks a systematic understanding of the relationship between gut microbial diversity and specific bone pathological states, as well as the precise mechanisms by which microbes regulate bone health.

Bibliometrics is a quantitative research method aimed at systematically analyzing the distribution, growth, and developmental trends of academic literature. Utilizing bibliometrics, scholars can gain a clearer perception of a field’s current research landscape, prominent research areas, and prospective research directions.

Regrettably, there are currently no bibliometric studies that have explored the relationship between the gut microbiota and osteoporosis. Given the context, this article employs bibliometric analysis to holistically assess existing research on the relationship between the gut microbiota and osteoporosis, aiming to identify research trends, key findings, existing gaps, and potential future directions.

## 2 Materials and methods

### 2.1 Search strategy

**FIGURE 1:**
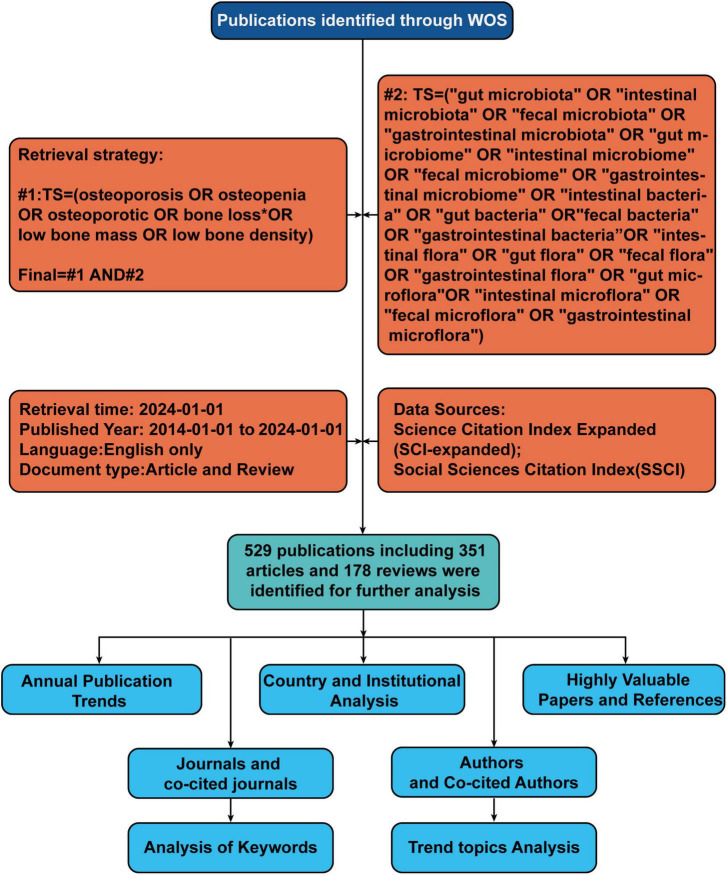
Flowchart of literature identification and analysis process. TS, topic; WoS, Web of Science.

We selected the Web of Science Core Collection (WoSCC) database to conduct a literature search on 01/03/2024. The search terms is as follows: #1: TS = (osteoporosis OR osteopenia OR osteoporotic OR bone loss* OR Low bone mass OR low bone density), #2: TS = (“gut microbiota” OR “intestinal microbiota” OR “fecal microbiota” OR “gastrointestinal microbiota” OR “gut microbiome” OR “intestinal microbiome” OR “fecal micro-biome” OR “gastrointestinal microbiome” OR “intestinal bacteria” OR “gut bacteria” OR “fecal bacteria” OR “gastrointestinal bacteria” OR “intestinal flora” OR “gut flora” OR “fecal flora” OR “gastrointestinal flora” OR “gut microflora” OR “intestinal microflora” OR “fecal microflora” OR “gastrointestinal microflora”), final = #1 AND #2. LA = (English), and the type of documents was set to “articles” and “review.”. The publication period was specified as 01/01/2014 to 30/03/2024. Following the initial retrieval, we screened the titles and abstracts to confirm the eligibility of the articles based on predefined inclusion and exclusion criteria. The flowchart of the screening process is shown in Figure 1.

### 2.2 Data analysis

Leveraging the capabilities of VOSviewer (version 1.6.18), a bibliometric analysis software of substantial renown (13), we facilitated the generation of visualizations representing cooperative, co-citation, and co-occurrence networks. The analyses conducted in this study utilizing VOSviewer encompassed co-occurrence analysis of keywords, nations, journals and co-cited journals, authors, and co-cited authors, as well as institutions. In the label view used for these visualizations, the colors of the nodes represent different clusters or groups of items (such as institutions, authors, or journals) that are more closely related to each other within the same cluster than to those in other clusters (14).

We also engaged CiteSpace (version 6.1. R1), an alternative software for bibliometric analysis and visualization (15), devised by Professor Chen Meichao of Drexel University. With the assistance of CiteSpace, we established dual map overlay visualizations of journals pertinent to this inquiry and pinpointed references and keywords exhibiting high citation bursts.

We used the R package “Bibliometrix” (version 3.2.1)^1^ to illustrate the annual publication volume trends in this research field and the publication output of various countries, which not only showcases the academic output of these countries but also elucidates the state of international collaboration among them. Subsequently, we created trend graphs of cumulative publication volumes for the top 10 institutions and journals, which further revealed the influence of major institutions and journals within the field. Moreover, we conducted a detailed analysis of trend topics using the “Bibliometrix” package.

### 2.3 Procedures for analysis

Full records and cited references of the retrieved articles were downloaded from the WoSCC database and saved as.txt format for below analysis.

#### 2.3.1 R package “Bibliometrix”

**FIGURE 3:**
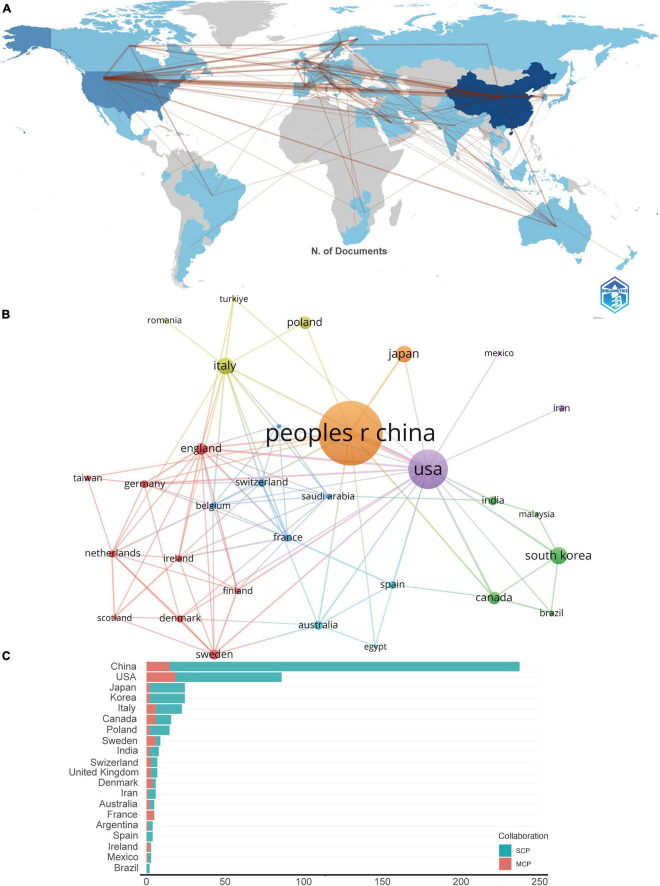
**(A)** The geographical network map. **(B)** The overlay visualization map of country co-authorship analysis conducted by VOSviewer. **(C)** TOP 20 corresponding author’s countries that produced the largest number of literature. SCP, single country publications; MCP, multiple country publications.

**FIGURE 4:**
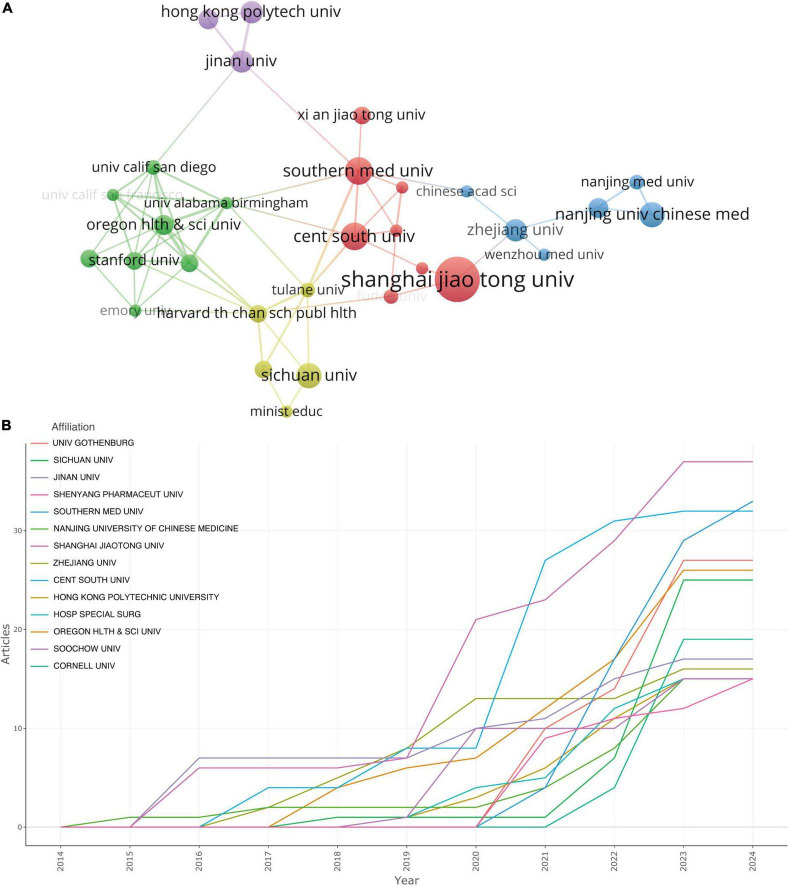
**(A)** The visualization of institutions cooperation networks based on VOSviewer. **(B)** Top 14 institutions’ production over time.

**FIGURE 5:**
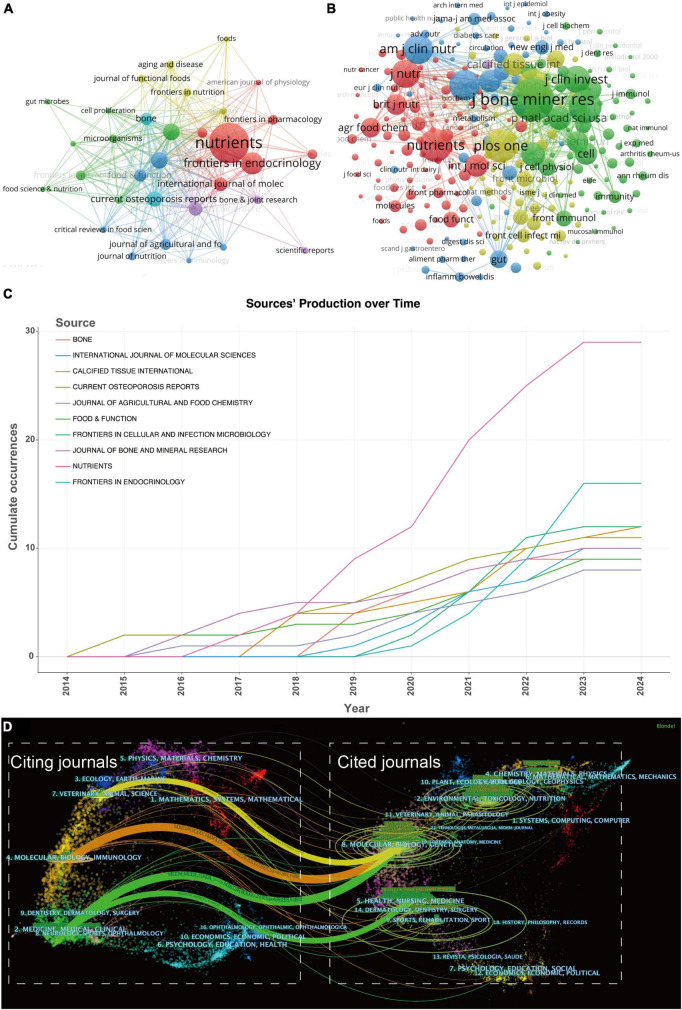
**(A)** The visualization of journals cooperation networks based on CiteSpace and **(B)** network visualization of co-cited journals based on VOSviewer. **(C)** Top 10 journals’ production over time. **(D)** The dual-map overlay of journals related to gut microbiota-osteoporosis. The overlay segments into two main areas: journals citing others on the left, and journals being cited on the right, connected by a trajectory curve representing citation paths. Ellipses in the diagram denote the publication volume of each journal, with the ellipse’s width indicating the diversity of contributing authors and its height reflecting the total number of articles published by the journal.

In utilizing the “Bibliometrix” package in R Studio for bibliometric analysis, the process begins with executing the biblioshiny () function to upload data via a web interface. For collaborative mapping among countries, set the parameters to a minimum of three connections and an edge size of 2.1 (Figure 3A). In the visualization of the corresponding author’s countries, set the number of countries to 20 (Figure 3C). The analysis then focuses on the top 14 institutions by publication volume, outlined in Figure 4B, and extends to the top 10 journals, depicted in Figure 5C. Lastly, to discern trending topics, adjust settings for a word frequency threshold of five and select three significant words per year, enabling an insightful delineation of research trends (Figure 8A).

#### 2.3.2 VOSviewer

**FIGURE 6:**
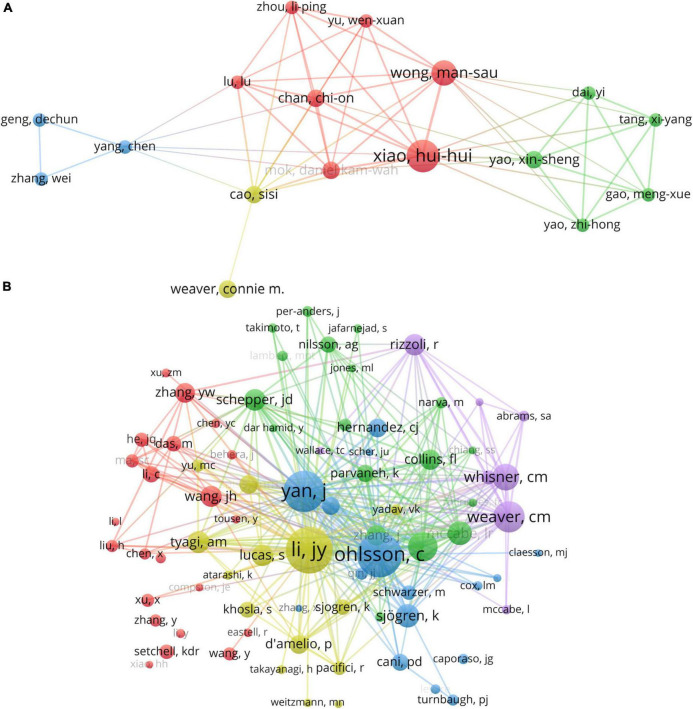
**(A)** The visualization of authors and **(B)** co-cited authors cooperation networks based on VOSviewer.

In the visualization of country cooperation relationships, we set a threshold of a minimum of 5 publications, resulting in 21 countries (out of 61) meeting the criteria (Figure 3B). In the analysis of institutional cooperation networks, out of 881 institutions, 45 had a publication count of at least 5 (Figure 4A). For the journal cooperation network, setting a threshold of at least 3 publications identified 37 journals (out of a total of 298) that qualified (Figure 5A). The co-cited journal network visualization used a minimum of 30 citations as a threshold, with 266 journals (out of 4,464) meeting the standard (Figure 5B). In the visualization of the author and co-cited author collaboration networks, we set thresholds of a minimum of 3 publications per author and 30 citations per author, respectively. The findings show that among 3,166 authors, only 25 satisfied the publication threshold (Figure 6A), while among 21,680 co-cited authors, 78 met the citation threshold (Figure 6B). For keyword co-occurrence analysis, a threshold of at least 5 co-occurrences was set, with 230 keywords (from a total of 2,606) meeting the standard (Figure 8B), and an overlay visualization of keywords was conducted, see Figure 8C.

#### 2.3.3 CiteSpace

During our analysis with CiteSpace software, we applied the following selection criteria: G-index set to 25; Link Retaining Factor (LRF) at 3.0; Look Back Year (LBY) of 5 years; and the percentage of marked nodes at 1.0%.

**FIGURE 7:**
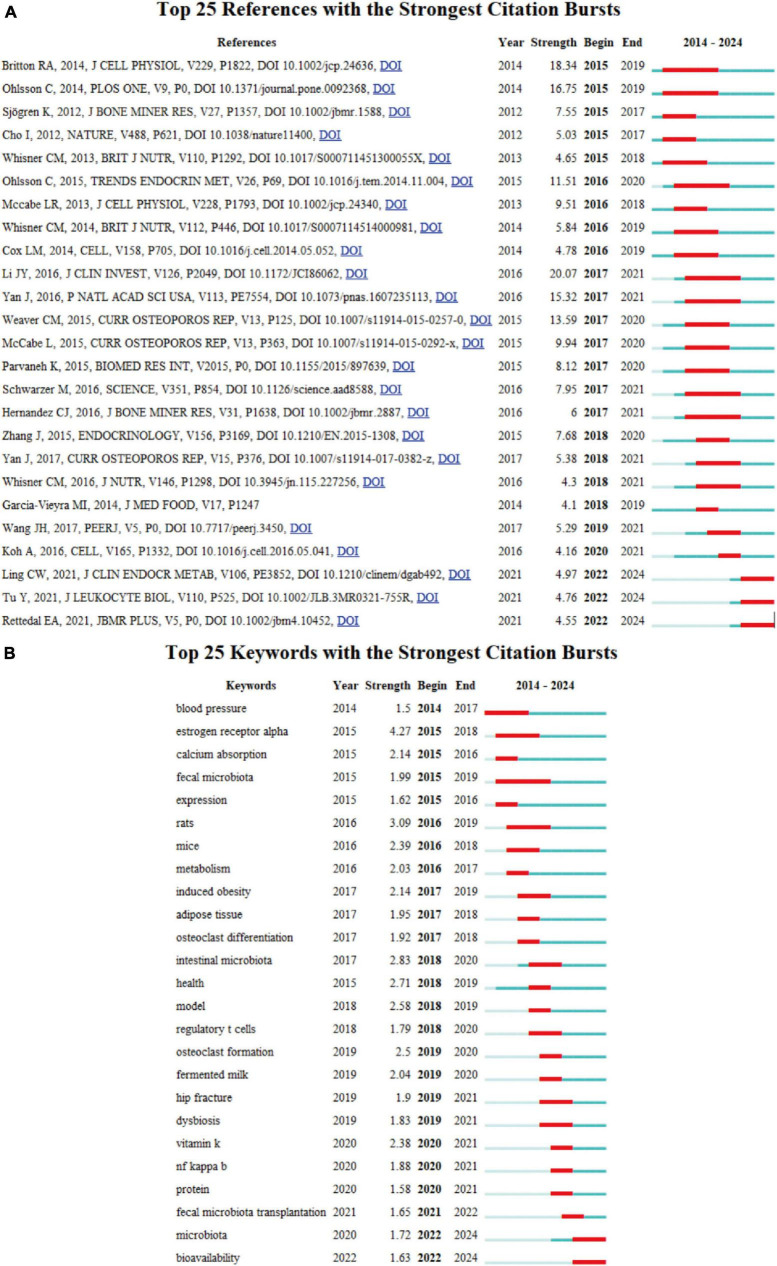
**(A)** Top 25 references with strongest citation bursts of publications regarding gut microbiota in osteoporosis. **(B)** Top 25 keywords with the strongest citation bursts based on CiteSpace.

For the burst analysis of references (Figure 7A) and the strong burst analysis of keywords (Figure 7B), we configured a specific detection model: f(x) = αe^–α^ *^x^*, α_1_/α_0_ = 0.2, α_*i*_/_α__*i*–1_ = 0/2; The Number of States = 2; ү = 0.2; Minimum Duration = 2.

## 3 Results

### 3.1 Annual publication trends

**FIGURE 2:**
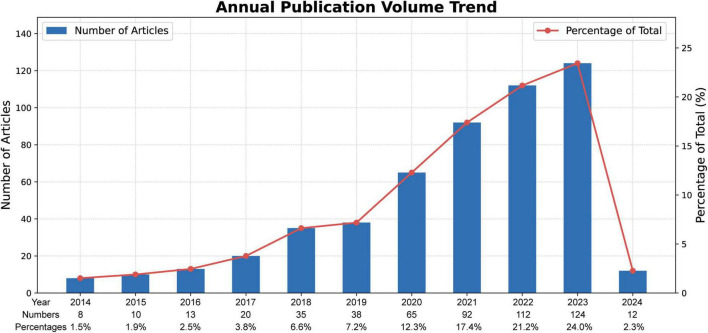
Annual outputs of publications regarding gut microbiota in osteoporosis field.

Considering the yearly increase in publication numbers, the entire period can be divided into two phases: Phase I (2014–2019), and Phase II (2020–2024). As shown in Figure 2, the number of publications in Phase I was relatively low, with an average annual publication count of about 20.6, representing the initial stage of research on gut microbiota- osteoporosis. Entering Phase II, the number of publications began to increase significantly, with an average annual publication count of 81, marking a substantial rise compared to Phase I. This trend demonstrates the growing recognition among scholars of the significance of gut microbiota in osteoporosis.

### 3.2 Country and institutional analysis

A total of 881 institutions, and 61 nations have contributed to this collective body of literature. As depicted in the geographical network map of Figure 3A, the top 10 contributors hail from diverse regions encompassing Asia, Europe, and North America. Table 1 discloses that a predominant portion of publications originates from China (253) and USA (111), collectively accounting for a commanding 68.8% of the total global publications. Hot on their heels are South Korea (*N* = 28, 5.29%), Italy (*N* = 27, 5.10%), and Japan (*N* = 27, 5.10%).

**TABLE 1:** Top 10 countries and institutions on research of gut microbiota in osteoporosis field.

Rank	Country	Articles	Citations	Rank	Institution	Counts
1	China	253	3,883	1	Shanghai Jiao Tong University	37
2	USA	111	3,537	2	Southern Medical University	33
3	South Korea	28	573	3	Central South University	32
4	Italy	27	769	4	University of Gothenburg	27
5	Japan	27	757	5	Nanjing University Chinese Medicine	26
6	Canada	17	350	6	Sichuan University	25
7	Poland	17	222	7	Cornell University	19
8	England	15	621	8	Jinan University	17
9	Sweden	11	391	9	Hong Kong Polytechnic University	16
10	Australia	10	256	10	Zhejiang University	15

Further, Table 1 accentuates that the publications from China boast the highest cumulative citation frequency (3,883), followed distantly by those from USA (3,537), Italy (769), and Japan (757). In Figure 3B, lines are utilized to represent the frequency of international academic collaboration, with the node’s size signifying each country’s publication tally. The visual representation in Figure 3B underscores a thriving international research collaboration landscape, with China, the United States, and Japan engaging in dynamic cooperative efforts. A close collaborative synergy between South Korea and Canada is also discernible.

Although China leads in publication volume by a significant margin compared to other countries, the proportion of its multi-country publications (MCP) is relatively low in comparison to its domestic research output, as shown in Figure 3C. This suggests that in this field of research, there is a notable lack of academic collaboration between the China and other countries.

We employed VOSviewer to conduct a visual analysis of the 881 institutions incorporated in this study, as portrayed in Figure 4A. Figure 4A offers a graphical delineation of the inter-institutional collaboration network, accentuating the strong ties that exist among a diverse array of institutions. As suggested by Figure 4A’s visualization, considerable collaboration is apparent between Shanghai Jiao Tong University and Zhejiang University, as well as between Southern Medical University and Central South University. However, it is pivotal to note that these collaborations principally take place within each institution’s home country, revealing a striking paucity of vibrant academic collaborations between institutions from disparate nations.

Figure 4B, alongside Table 1, ranks the top institutions by their contributions to the literature. Shanghai Jiao Tong University leads the cadre with 37 publications, closely followed by Southern Medical University and Central South University with 33 and 32 publications, respectively. Notably, the disparity in publication volume across these institutions does not emerge as significant.

Figure 4B showcases a promising incline in the annual publication output from the top 14 institutions in recent years. Nanjing University of Chinese Medicine blazed the trail by being the first to contribute to this field, but its cumulative document count appears to have plateaued starting from 2017. Conversely, although Shanghai Jiao Tong University joined the field later, it has witnessed a swift escalation in publication output beginning from 2019.

### 3.3 Journals and co-cited journals

Harnessing the capabilities of VOSviewer, we curated a visual representation of journals and co-cited journals within this research realm. Our dataset includes a total of 246 journals, and we incorporated the top 33 journals with a minimum publication count of 4 (Figure 5A); the size of the nodes represents the publication volume of each journal. Figure 5B unfurls a network map of co-cited journals, featuring those commanding a minimum of 25 citations. As explicated in Figure 5B, 312 co-cited journals were displayed, reflecting the aggregate link strength. The five most frequently co-cited journals, exhibiting the most formidable total link strength (TLS), comprised: *J Bone Miner Res* (TLS = 85,399), *Nutrients* (TLS = 58,480), *Nature* (TLS = 57,100), *PLoS one* (TLS = 55,021), and *Bone* (TLS = 54,481) (refer to Table 2).

**TABLE 2:** Top 10 journals and co-cited journals for gut microbiota in osteoporosis.

Rank	Journal	Counts	IF	Q	Co-cited journal	Citations	TLS
1	Nutrients	29	6.71	Q1	J Bone Miner Res	1,096	85,399
2	Frontiers in Endocrinology	16	6.05	Q1	PLoS One	727	55,021
3	Frontiers in Cellular and Infection Microbiology	12	6.07	Q1	Nature	725	57,100
4	Calcified Tissue International	12	4.20	Q1	Nutrients	713	58,480
5	Current Osteoporosis Reports	11	5.16	Q2	Bone	694	54,481
6	Journal of Bone and Mineral Research	10	6.39	Q1	Osteoporosis Int	649	50,281
7	International Journal of Molecular Sciences	10	5.61	Q1	Am J Clin Nutr	607	52,133
8	Bone	9	4.62	Q2	J Nutr	565	46,210
9	Food & Function	9	5.98	Q1	J Clin Invest	531	45,583
10	Journal of Agricultural and Food Chemistry	8	6.12	Q1	Sci Rep-UK	427	33,344

Local citations, deduced from the reference list, afford insight into their localized impact, whereas total citations mirror wider interest across various disciplines. Within this ranking, *J Bone Miner Res* commandeered the list with 1,096 citations, followed by *PLoS One* with 727 citations, and *Nature* with 725 citations (as illustrated in Table 2). This clearly indicates a high proportion of high-caliber publications within these journals. Clearly, these journals are high-quality international publications that provide support for gut microbiota-osteoporosis research.

Figure 5C sketches the annual outputs of the top 10 journals spanning from 2014 to 2024. The publication volume in *Nutrients* has experienced a steep ascent in recent years. Conversely, the publication growth in *Journal of Agricultural and Food Chemistry* has been relatively placid. Table 2 catalogs the top 10 most productive and co-cited journals incorporated in this inquiry. *Nutrients* (impact factor = 6.71, 2024) surfaced as the preeminent publisher, boasting 29 publications. Further, there were 16 publications in *Frontiers in Endocrinology* (IF = 6.05, 2024), 12 publications in *Frontiers in Cellular and Infection Microbiology* (IF = 6.07, 2024) and *Calcified Tissue International* (IF = 4.20, 2023). Eight of the top 10 journals fell under the Q1 JCR region.

In CiteSpace, the dual-map overlay technique provides researchers with a macroscopic view of the cross-disciplinary interactions and citation relationships among scholarly articles. This visualization is composed of two parts: one represents the disciplines of the citing articles, and the other represents the disciplines of the cited articles, each displayed on the left and right maps, respectively. The labels on these maps identify the journals or research areas involved. The paths from citing to cited articles reveal the flow of knowledge between disciplines. The color and thickness of these paths indicate the citation intensity and the time frame, assisting researchers in quickly identifying academic trends and impacts over different periods. Thus, the dual-map overlay not only serves as a bridge for communication between various disciplines but also highlights key areas in academic dissemination, providing a powerful tool for exploring trends, predicting hot research topics, and fostering potential interdisciplinary collaborations (16, 17).

Utilizing CiteSpace, we crafted a dual map overlay of journals pertaining to the role of gut microbiota in osteoporosis, as illustrated in Figure 5D. Clusters residing on the left of the orange line designate citing journals, whereas the cluster to the right of the orange trajectory signifies co-cited journals. The principal path (the yellow, orange, and one green paths) reveals that articles emanating from the realms of molecular/biology/genetics are primarily cited by researchers engaged in veterinary/animal/science journals, molecular/biology/immunology journals and medicine/medical/clinical journals. Furthermore, the other green path indicates that articles originating from the spheres of health/nursing and medicine are mainly cited by researchers involved in medicine and clinical journals. The outcomes from the dual-map overlay of journals may suggest that the current gut microbiota – osteoporosis research is zeroed in on molecular medical and clinical aspects.

### 3.4 Authors and co-cited authors

In the exploration of gut microbiota in osteoporosis, 3,166 researchers participated. The top 10 contributors collectively produced 79 publications, representing approximately 14.9% of the total output within this field (Table 3). Ohlsson C and Hernandez CJ emerged as the most productive authors, with 9 publications (Table 3). The H-index, a metric designed to quantify a scholar’s impact who has authored H papers each garnering at least H citations, was employed to appraise the influence of the scientific investigations. As evidenced in Table 3, Li YJ distinguished himself as the author boasting the highest H-index, followed by Hernandez CJ et al. Anchored in the understanding of the brain-gut-bone axis and its effects on bone metabolism, Li YJ’s highest impact factor paper (published in *Crit Rev Food Sci Nutr*, IF = 11.20) probes into the potential influence of probiotics and prebiotics on osteoporosis (18). This extensive exploration touches on various facets, including the regulation of gut metabolites, the integrity of the intestinal epithelial barrier, and the critical roles played by neuromodulation, immune regulation, and endocrine regulation. By doing so, the article shines a light on a promising and innovative approach toward the prevention and treatment strategies of osteoporosis in the future.

**TABLE 3:** Top 10 authors and co-cited authors on research of gut microbiota in osteoporosis field.

Rank	Author	Counts	H-index	Co-cited Author	Citations	Total link strength
1	Ohlsson C	9	5	Li YJ	205	4,724
2	Hernandez CJ	9	6	Ohlsson C	197	4,266
3	Li YJ	8	6	Yan J	180	4,059
4	Rui YF	8	5	Weaver CM	134	2,890
5	Zhang YW	8	6	Britton RA	125	2,692
6	Sjogren K	8	4	Whisner CM	112	2,573
7	Xiao HH	8	4	Mccabe LR	99	2,253
8	Cao MM	7	4	Sjogren K	97	1,803
9	Fu LG	7	5	Tyagi AM	91	2,243
10	Parameswaran N	7	6	Schepper JD	90	2,295

VOSviewer provides a visualization of the interconnections among authors, as exhibited in Figure 6A. There exists a profound collaboration between Xiao HH and Wong MS as well as Chan CO. Similarly, a dynamic partnership is observed between Yao, Xin-Sheng and Yao, Zhi-Hong. Co-citation analysis scrutinizes the association among items based on their co-citation frequency. Deploying VOSviewer, a totality of 153 authors, each with a minimum citation count of 20, were evaluated, as delineated in Figure 6B.

As expounded in Table 3, Li YJ emerges as the most frequently co-cited author (co-citation = 205), succeeded by Ohlsson C (co-citation = 197), and Yan J (co-citation = 180). Out of the 17,853 co-cited authors, six scholars received more than 100 co-citations. Ohlsson C’s most cited article reviews the impact of the gut microbiota on bone mass (19). The research indicates that the gut microbiota regulates bone mass by modulating the host immune system. Moreover, dietary and environmental factors can influence the composition of the gut microbiota, thus affecting bone metabolism. The authors put forward the idea that the gut microbiota could serve as a novel therapeutic target for treating osteoporosis. This groundbreaking work is the first to propose that the gut microbiota could be an innovative approach toward managing osteoporosis and preventing fractures.

### 3.5 Hotspots investigation

#### 3.5.1 Highly valuable papers

To evaluate the publications’ influence on osteoporosis research, we evaluated local citations. Table 4 lists the top 10 co-cited documents on gut microbiota studies in osteoporosis. The most frequently cited paper, “Sex steroid deficiency–associated bone loss is microbiota dependent and prevented by probiotics,” amassed 143 citations (20). This article investigates the relationship between the gut microbiota, sex steroid deficiency-induced bone loss, and the potential therapeutic role of probiotics. The research shows that sex steroid deficiency results in increased gut permeability, expanded Th17 cells, and elevated levels of osteoclastogenic cytokines in the small intestine and bone marrow. These changes were not observed in germ-free mice, suggesting the necessity of gut microbiota-induced alterations in intestinal permeability and inflammatory responses for sex steroid deficiency-induced trabecular bone loss.

**TABLE 4:** The top 10 documents with the most local citations.

Rank	Title	LC	Journal	IF	PY	Author
1	Sex steroid deficiency–associated bone loss is microbiota dependent and prevented by probiotics	143	J Clin Invest	15.9	2016	Li JY
2	Diversity analysis of gut microbiota in osteoporosis and osteopenia patients	78	PeerJ	2.7	2017	Wang JH
3	*Lactobacillus reuteri* reduces bone loss in older women with low bone mineral density: a randomized, placebo-controlled, double-blind, clinical trial	66	J Intern Med	11.1	2018	Nilsson AG
4	Effects of the gut microbiota on bone mass	65	Trends Endocrinol Metab	10.9	2015	Ohlsson C
5	Gut microbiota alterations associated with reduced bone mineral density in older adults	62	Rheumatology	5.5	2019	Das M
6	Probiotics (*Bifidobacterium longum*) increase bone mass density and upregulate Sparc and Bmp-2 genes in rats with bone loss resulting from ovariectomy	60	Biomed Res Int	0.0	2015	Parvaneh K
7	Gut microbiota composition and bone mineral loss—epidemiologic evidence from individuals in Wuhan, China	60	Osteoporos Int	4.0	2019	Li C
8	Gut microbiota and metabolite alterations associated with reduced bone mineral density or bone metabolic indexes in postmenopausal osteoporosis	57	Aging	5.2	2020	He JQ
9	Diet, gut microbiome, and bone health	56	Curr Osteoporos Rep	4.3	2015	Weaver CM
10	Involvement of the gut microbiota and barrier function in glucocorticoid-induced osteoporosis	48	J Bone Miner Res	6.2	2020	Schepper JD

Moreover, references which garner widespread citation over time within a particular subject are identified as references with citation bursts. Serving as a valuable metric, these burst citations highlight references that have captured academic interest within a specified field during a certain timeframe. In this investigation, CiteSpace pinpointed the top 25 references bearing the most significant citation bursts, displayed in Figure 7A. Among these, Li JY’s article, which was mentioned above as the most frequently cited paper (20), held the highest rank (strength = 20.07). Ranking secondly, the research article by Britton et al., titled “Probiotic *L. reuteri* Treatment Prevents Bone Loss in a Menopausal Ovariectomized Mouse Model,” published in the *Journal of Cellular Physiology*, investigates the effects of the probiotic *Lactobacillus reuteri* ATCC PTA 6475 on bone health in a model of menopause-induced osteoporosis. The study concludes that *L. reuteri* treatment suppresses bone resorption and loss associated with estrogen deficiency, suggesting that it may be a cost-effective approach to mitigate post-menopausal bone loss. This underscores the importance of gut microbiota in bone health and the potential of probiotics as a therapeutic strategy for osteoporosis (21).

R package “Bibliometrix” identified the top 10 most co-cited references, which are exhibited in Table 5. The two most-cited references are the same as those mentioned above, written by Li JY and Britton RA, respectively, and will not be reiterated here. In the third cited article authored by Yan J, they demonstrate that the resident gut microbiota not only stimulate bone formation but also resorption, with prolonged exposure to microbiota resulting in overall skeletal growth (22). The microbiota triggers the hormone insulin-like growth factor 1 (IGF-1), a key player in bone growth and remodeling. SCFAs, generated when microbiota ferment fiber, also stimulate IGF-1, hinting at a mechanism through which microbiota can impact bone health.

**TABLE 5:** The top 10 most co-cited references in the field of gut microbiota in osteoporosis.

Rank	Title	TC	Journal	IF	PY	Author
1	Sex steroid deficiency–associated bone loss is microbiota dependent and prevented by probiotics	118	J Clin Invest	19.4	2016	Li, JY
2	Probiotic *L. reuteri* treatment prevents bone loss in a menopausal ovariectomized mouse model	98	J Cell Physiol	6.5	2014	Britton RA
3	Gut microbiota induce IGF-1 and promote bone formation and growth	89	PANS	12.7	2016	Yan J
4	The gut microbiota regulates bone mass in mice	76	J Bone Miner Res	6.3	2012	Sjögren K
5	Probiotics protect mice from ovariectomy-induced cortical bone loss	70	PLoS One	3.7	2014	Ohlsson C
6	Short-chain fatty acids regulate systemic bone mass and protect from pathological bone loss	69	Nat Commun	17.6	2018	Lucas S
7	Diversity analysis of gut microbiota in osteoporosis and osteopenia patients	63	PeerJ	3.0	2017	Wang JH
8	The microbial metabolite butyrate stimulates bone formation via T regulatory cell-mediated regulation of WNT10B expression	54	Immunity	43.4	2018	Tyagi AM
9	*Lactobacillus reuteri* reduces bone loss in older women with low bone mineral density: a randomized, placebo-controlled, double-blind, clinical trial	51	J Intern Med	13.0	2018	Nilsson AG
10	Gut microbiota alterations associated with reduced bone mineral density in older adults	50	Rheumatology	7.04	2019	Das M

In essence, these significant studies primarily address the function of the gut microbiome in bone health, including discussions on how the composition and metabolites of the gut microbiome affect bone mass and bone mineral density, with special emphasis on the immune system and inflammation. Furthermore, some papers also explored how diet and probiotics (such as *L. reuteri*) affect bone health by acting on the gut microbiota. The research areas covered in these papers likely reflect the evolving hotspots in the field of gut microbiota and osteoporosis studies. Further analysis on this will be conducted in the discussion section of our paper.

#### 3.5.2 Analysis of keywords

Keywords reflect the core or the main points the author wishes to express in an article. Therefore, keyword analysis in bibliometrics allows for exploration of hot topics and trends in the field. The keyword co-occurrence analysis facilitates the prompt identification of research focal points within a given area. Table 6 enumerates the 20 terms exhibiting the highest frequency within this field. The leading four keywords from the co-occurrence analysis include: gut microbiota (285 occurrences), osteoporosis (262 occurrences), inflammation (69 occurrences), and health (69 occurrences), all key terms associated with thematic research. It is noteworthy that “inflammation” and “probiotics” have appeared over 50 times, possibly indicating that investigations into how dysbiosis-induced inflammation in the gut microbiome contributes to osteoporosis, along with the potential of probiotics to manage and treat osteoporosis by altering the gut microbiota, are likely major research priorities in this area.

**TABLE 6:** Top 20 keywords on research of gut microbiota in osteoporosis.

Rank	Keywords	Occurrences	Rank	Keywords	Occurrences
1	Gut microbiota	285	11	Bone loss	52
2	Osteoporosis	262	12	Intestinal microbiota	47
3	Inflammation	69	13	Double-blind	47
4	Health	69	14	Microbiota	45
5	Mineral density	64	15	Obesity	44
6	Bone-mineral density	64	16	Chain fatty-acids	42
7	Gut microbiome	61	17	Microbiome	40
8	Probiotics	54	18	Women	38
9	Bone	53	19	Oxidative stress	37
10	Postmenopausal women	52	20	Differentiation	35

**FIGURE 8:**
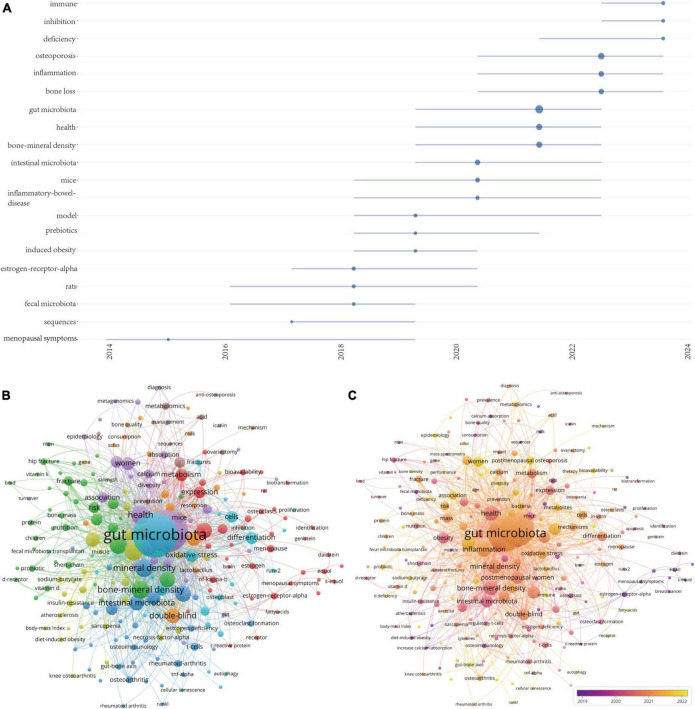
**(A)** Visualization map of trend topics analysis. **(B)** Keywords co-occurrence network and **(C)** overlay visualization of the keywords network based on VOSviewer.

The keyword burst analysis by CiteSpace (Figure 7B) and the trend topic analysis by the R package “Bibliometrix” (Figure 8A) allow us to understand the hot research areas of particular periods and the most recent trends in this research domain. In the keyword burst analysis, the keyword “estrogen receptor alpha” had the highest burst strength (strength = 4.27), with a burst period from 2015 to 2018. The term “fecal microbiota” exhibited the longest burst duration, spanning 4 years. Additionally, keywords emerging in the past 3 years, including fecal microbiota transplantation (strength = 1.65), microbiota (strength = 1.72), and bioavailability (strength = 1.63), represent emerging fields.

Using a minimum co-occurrence threshold of 5, we included 230 keywords in a cluster analysis using VOSviewer. As depicted in Figure 8B, the blue cluster is very clear, with keywords in this cluster primarily including “probiotics,” “t-cells,” “chain fatty-acids,” “mineral density,” and “TNF-α.” Figure 8C displays high-frequency keywords in an overlay graph, with the colors indicating the average publication year. Combining this with the trend topic map in Figure 8A, it is evident that scholars have been actively investigating the role of the interaction between gut microbiota and the immune system in the progression of osteoporosis, as well as the inflammation initiated by the gut microbiota.

In summary, the keyword analysis section clearly highlights that dysbiosis-induced inflammation is a primary area of focus in the study of gut microbiota-osteoporosis. This focus is evidenced by the prevalent occurrence of terms such as “inflammation” and “probiotics.” Furthermore, the term “fecal microbiota transplantation” emerges as a noteworthy area of interest, especially noted for its increasing relevance in recent years. This suggests that fecal microbiota transplantation could be a promising future research direction, potentially effective in modulating gut microbiota to combat osteoporosis.

## 4 Discussion

Using the WOSCC database, this study searched for literature related to the gut microbiota and osteoporosis from 2014 to 2024. Subsequently, several bibliometric analysis tools were utilized to visually analyze the literature, to further understand the research status of gut microbiota in osteoporosis over the past decade, and to explore the research hotspots and frontiers in this field.

We summarized the top co-cited documents and references with citation bursts, finding that these highly valuable papers primarily focus on how the composition and metabolites of the gut microbiome affect bone mass and bone mineral density, with a particular emphasis on the immune system and inflammation.

Additionally, by analyzing the frequency of keywords, overlay displays, and burst detection results, we discovered that research on inflammation caused by dysbiosis in osteoporosis is a research hotspot and frontier in this field.

### 4.1 Dysbiosis-induced inflammation: central to the pathophysiology of osteoporosis

The gut microbiota profoundly and subtly impacts host bone health (23), both directly [for example, through microbe-associated molecular patterns (MAMP) or pathogen-associated molecular patterns (PAMP)] and indirectly (for instance, via metabolite production), by modulating innate pro-inflammatory or anti-inflammatory reactions (24).

Eubiosis, colloquially known as a “healthy microbiota,” is recognized as a crucial factor in preserving the physiological and metabolic integrity of the organism. Traditionally, eubiosis is conceived as a harmonious balance within the gut microbiome ecosystem, characterized by a predominance of beneficial bacterial species (25). These beneficial microbiota consortia reinforce intestinal epithelial barrier integrity through direct, cooperative mechanisms. Furthermore, they shape the host’s immune landscape indirectly by generating a variety of essential metabolites.

Studies indicate that SCFAs are the most significant metabolites known to positively affect Treg cells, particularly butyrate, which enhance Treg cell differentiation and function (26). This is achieved by promoting the expression of relevant genes and by inhibiting the activity of histone deacetylases. Moreover, indole-3-acetic acid (IAA) and indole-3-propionic acid (IPA), metabolites produced by the gut microbiota from tryptophan, can promote the differentiation of Treg cells as well, but through activating the aryl hydrocarbon receptor (AhR) (27). Consequently, an increase in Treg cells triggers the release of anti-inflammatory cytokines and reduces inflammatory signaling pathways, such as NF-κB, thereby enhancing intestinal immune tolerance and maintaining an anti-inflammatory milieu (28).

Contrary to eubiosis, “dysbiosis” denotes alterations in the composition and function of the primary microbial communities, linked to the emergence of various diseases (25).

**FIGURE 9:**
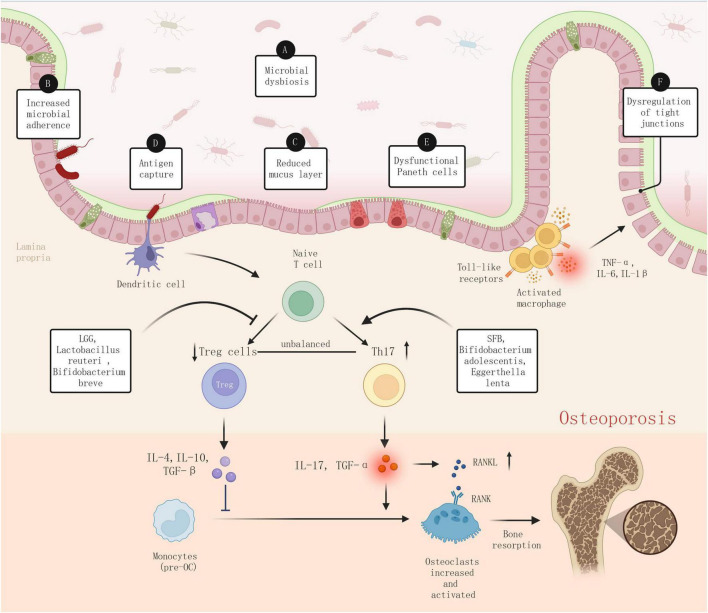
Dysbiosis-induced inflammation in pathophysiology of osteoporosis.

Dysbiosis in the gut microbiota disrupts the “functional balance” between pro-inflammatory and anti-inflammatory microbes, altering intestinal immunity and biasing the immune system toward a pro-inflammatory response (Figure 9). This alteration affects the differentiation of naive CD4+ T cells. In particular, specific bacteria, including segmented filamentous bacteria (SFB), *Bifidobacterium adolescentis*, and *Eggerthella lenta*, are known to augment Th17 cells (29). This leads to an increase in the secretion of pro-inflammatory cytokines, such as IL-17 and TNF-α from Th17 cells, further promoting inflammatory responses and bone resorption (30). Conversely, certain bacterial species, including *Lactobacillus rhamnosus* GG (LGG), *L. reuteri*, and *Bifidobacterium breve*, can positively impact the abundance and function of Treg cells (29). However, the reduction of these beneficial bacterial species can inhibit the differentiation of regulatory T cells (Tregs), leading to a decrease in the secretion of anti-inflammatory cytokines derived from Tregs, such as IL-4, IL-10, and TGF-β. This ultimately promotes osteoclastogenesis and inhibits bone formation. The dysbiosis-induced alteration in the Th17/Treg balance reduces the host’s immunosuppressive capacity, which exacerbates the inflammatory state of the intestinal microenvironment and directly impacts bone metabolism.

As mentioned above, dysbiosis can also be defined as alterations in the composition of the primary microbial communities, typically characterized by a reduction in beneficial bacteria and/or an increase in harmful bacteria. This reduction in beneficial microbes can not only directly compromise the integrity of connections between intestinal epithelial cells, diminishing the thickness and quality of the gut mucosal mucus layer, thus reducing barrier function and enhancing intestinal permeability, a condition commonly referred to as “leaky gut” (31). Furthermore, the proliferation of pathogenic bacteria and their toxins (such as lipopolysaccharide, LPS) can stimulate intestinal mucosal immune cells: dendritic cells (DCs) in the intestine can capture and process antigens, activating T cells, and promoting the differentiation of Th17 cells (32). Macrophages then trigger inflammatory responses through pattern recognition receptors (such as Toll-like receptors), resulting in the production of excessive pro-inflammatory cytokines, such as TNF-α, IL-6, and IL-1β (33). These inflammatory factors, secreted by intestinal mucosal immune cells in response to pathogens and toxins, not only increase local intestinal inflammation but can also directly damage intestinal epithelial cells, leading to reduced expression of tight junction proteins (such as occludin and claudin), thereby further increasing intestinal permeability. Consequently, bacteria, toxins, and other foreign antigens can more easily enter the bloodstream, leading to a systemic inflammatory response.

The impact of inflammation on bone metabolism is well recognized (34, 35). Chronic systemic inflammation, through the activation of immune cells such as macrophages and T cells, leads to the production of inflammatory cytokines (TNF-α, IL-1, and IL-6) that directly influence bone metabolism. Specifically, TNF-α and IL-1 are known to promote the differentiation and maturation of osteoclast precursors, leading to increased bone resorption (36, 37). Additionally, IL-6 facilitates osteoclastogenesis by upregulating RANKL expression and drives bone immune reactions via the JAK/STAT signaling pathway, thus accelerating bone resorption (38). Moreover, IL-17 boosts osteoclast activity by fostering RANKL expression, thereby influencing bone resorption, and it may indirectly affect osteoblast function by inhibiting the Wnt/β-catenin signaling pathway (39). Furthermore, the inflammatory milieu amplifies the interaction between RANKL (a key osteoclast differentiation factor) and its receptor RANK, further enhancing osteoclast formation and activity (40).

Overall, the inflammation initiated by gut microbiota dysbiosis and the resulting immune disorder play a pivotal role in osteoporosis progression. Inflammatory responses triggered by dysbiosis lead immune cells, such as macrophages and dendritic cells, to release an array of inflammatory mediators, directly impacting bone metabolism through key pathways such as the NF-κB signaling pathway and the RANKL/OPG/RANK system, among others. Additionally, the inflammatory state from dysbiosis disturbs the dynamic equilibrium between Th17 and Tregs, not only facilitating osteoclast differentiation but also suppressing bone formation, thus laying the groundwork for osteoporosis development. These mechanisms highlight the critical importance of understanding the interplay between gut microbiota and the host immune system, suggesting that regulating this intricate network might provide novel prevention and treatment options for osteoporosis.

Among current drug treatments for osteoporosis, a range of options such as bisphosphonates, SERMs, PTH and its analogs, RANKL inhibitors, and Sclerostin inhibitors have been confirmed to effectively increase bone density, decelerate bone loss, and lower fracture risks (41, 42). Although these drugs offer viable treatments for osteoporosis, their application is faced with several limitations. These limitations range from potential safety issues with long-term use, such as jaw osteonecrosis and atypical femur fractures associated with bisphosphonates, to significant patient-to-patient variability in treatment outcomes. Additionally, the high costs of developing new drugs and an inadequate understanding of complex disease mechanisms present further challenges. Furthermore, existing treatments mainly target a single aspect of the pathological process and often fail to address the multifactorial etiology of osteoporosis, highlighting the need for more comprehensive treatment strategies.

### 4.2 Targeting gut microbiota: FMT and diet in osteoporosis treatment

**FIGURE 10:**
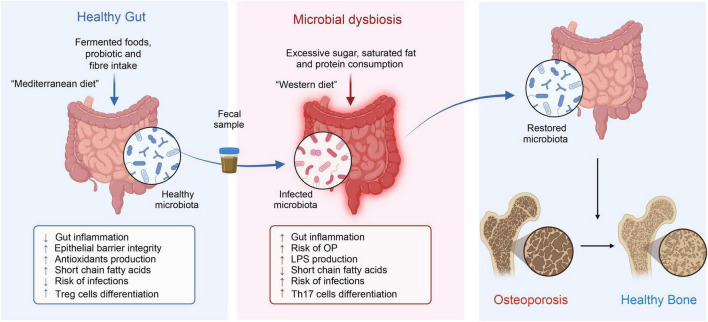
Fecal microbiota transplantation and diet in osteoporosis treatment.

Given the crucial role of gut microbiome dysbiosis and resultant inflammation in the pathogenesis of osteoporosis, focusing research on the gut microbiome as a treatment target for osteoporosis is not only a response to the limitations of current treatments but also a logical extension of a comprehensive understanding of osteoporosis’s complex pathophysiological mechanisms. Keywords with the strongest citation bursts, such as “fecal microbiota transplantation,” “fermented milk,” “protein,” “bioavailability,” “nf kappa b,” highlight that regulating the gut microbiome through methods like fecal microbiota transplantation or specific dietary patterns to control inflammation represents a promising future research direction for enhancing bone health and preventing or treating osteoporosis (Figure 10).

Diet has a significant impact on bone health throughout the entire lifespan and is a major determinant of the types and proportions of microbes in the host organism. Thus, when assessing the impact of changes in the gut microbiome on bone health, diet should be considered an important confounding factor (43).

Diet directly affects the gut microbiome, modifying its composition or metabolic outputs, potentially contributing to disease progression or maintaining bodily balance (44). A high-fiber diet rich in fruits, vegetables, legumes, and whole grains provides various polysaccharides and oligosaccharides indigestible by human enzymes. These dietary fibers act as prebiotics, specifically nourishing beneficial gut bacteria and promoting the production of SCFAs (such as butyrate, propionates, and acetates) (45). Fermented foods such as yogurt, kefir, sauerkraut, and kimchi are rich in live microorganisms that contribute to the increased diversity of the gut microbiota. By consuming fermented foods, one can increase the abundance of beneficial bacteria, improve gut barrier function, and have anti-inflammatory effects (46). The Mediterranean diet, enriched with vegetables, fruits, nuts, seeds, legumes, whole grains, fish, and olive oil, is associated with increased microbial diversity and the promotion of beneficial bacterial growth. This diet, rich in monounsaturated fats, polyphenols, and fiber, not only positively affects the gut microbiome but also enhances the production of SCFAs, thereby improving gut barrier function, and reducing inflammation as common benefits (47).

High-protein diets, especially those rich in animal proteins, alter the gut microbiome by increasing the abundance of bacteria capable of fermenting protein. Consequently, this could lead to the production of potentially harmful by-products, including ammonia, amines, and sulfides (48). Western diets, marked by high fat, high sugar, and low fiber, lead to a gut microbiota composition inclined toward increased inflammation levels and decreased diversity (49). Such dietary patterns commonly result in increased intestinal permeability (“leaky gut”) and systemic inflammation.

In recent years, fecal microbiota transplantation (FMT) has been widely used to treat a variety of diseases (50), including Crohn’s disease, metabolic syndrome (51), diabetes (52), and neurological disorders (53). Remarkably, FMT has also demonstrated considerable potential in treating osteoporosis (54). Unlike individual or mixed bacteria, FMT maximally retains the original diversity and quantity of active microbial communities, thereby enabling a quicker reinstatement of gut microbiota stability in osteoporosis patients (54). In a study by Zhang et al., transplanting the gut microbiota of children into ovariectomized (OVX) mice effectively prevented bone loss caused by ovariectomy and increased the bone strength of the mice. Moreover, 16S rRNA gene sequencing revealed that transplanting the fecal microbiota of children reversed the OVX-induced reduction in *Akkermansia* abundance, while direct supplementation with *Akkermansia* could prevent bone loss in OVX mice (54).

Yet, the choice of FMT donors, the presence of numerous harmful bacteria in the transplants, and the long-term outcomes of the treatment approach continue to cast doubt on its clinical safety (55, 56). Future research should focus on identifying specific microbial strains or consortia that have the most significant impact on bone metabolism, exploring the optimal timing and frequency of FMT for the best bone health outcomes, and understanding individual variations in response to FMT. Clinical trials that monitor the gut microbiota composition, bone density, and bone metabolism markers before and after FMT are essential to ascertain the effectiveness of this method in preventing or treating osteoporosis.

Research on various interventions targeting the gut microbiota is still in its initial stages, and we emphasize the necessity for future studies, particularly the need for more high-quality, large-scale, long-term clinical and mechanistic studies to validate the efficacy and safety of gut microbiota intervention measures.

## 5 Conclusion

Through a bibliometric analysis of literature on osteoporosis and gut microbiota published between 2014 and 2024, this paper investigates the impact of gut microbiome dysregulation and its associated inflammation on osteoporosis progression, a topic that has garnered international research interest in recent years. Additionally, our study delves into the potential of fecal microbiota transplantation or specific dietary interventions as promising avenues for future research.

Recent human studies have demonstrated a significant role of gut health in bone metabolism, highlighting the potential of the gut microbiota as a therapeutic strategy in osteoporosis. However, variations in the test environment, the genetic background of the host, and the sources of gut microbiota present major challenges in controlling variables in research. These factors contribute to the heterogeneity and some contradictory conclusions in current studies. As a result, transitioning gut microbiota research from basic studies to clinical trials and practical applications remains a challenge. A critical priority is to continue searching for effective gut microbial strains for the treatment of osteoporosis and to carefully evaluate their quality, safety, dosage, stability, and interactions with other drugs. Moreover, ongoing and future studies must rigorously validate these findings in larger human cohorts to establish a more definitive link between the gut microbiota and osteoporosis. We eagerly anticipate further high-quality research that sheds light on the intricate dynamics between the gut microbiota and osteoporosis, ultimately charting new courses for osteoporosis prevention and therapy.

## Data availability statement

The original contributions presented in this study are included in the article/supplementary material, further inquiries can be directed to the corresponding authors.

## Ethics statement

This study did not include any patient information. Thus, the requirement for ethics approval was waived.

## Author contributions

ZL: Writing – original draft. YH: Conceptualization, Writing – original draft. ZC: Data curation, Writing – original draft. CL: Methodology, Writing – original draft. BZ: Supervision, Writing – review & editing. XG: Investigation, Writing – review & editing. ZS: Data curation, Funding acquisition, Writing – review & editing. TW: Project administration, Visualization, Writing – review & editing.

## Footnotes

1
https://www.bibliometrix.org
